# Sonogenetics in the Treatment of Chronic Diseases: A New Method for Cell Regulation

**DOI:** 10.1002/advs.202407373

**Published:** 2024-11-03

**Authors:** Mingrui Zhu, Yan Fang, Yikang Sun, Shaoyue Li, Jifeng Yu, Bing Xiong, Congjian Wen, Boyang Zhou, Bin Huang, Haohao Yin, Huixiong Xu

**Affiliations:** ^1^ Department of Ultrasound Institute of Ultrasound in Medicine and Engineering Zhongshan Hospital Fudan University Shanghai 200032 P. R. China; ^2^ Department of Ultrasound, Huashan Hospital Fudan University Shanghai 200040 P. R. China; ^3^ Department of Medical Ultrasound, Center of Minimally Invasive Treatment for Tumor Shanghai Tenth People's Hospital Ultrasound Research and Education Institute Clinical Research Center for Interventional Medicine School of Medicine Tongji University Shanghai 200072 P. R. China; ^4^ Zhejiang Hospital Hangzhou 310013 P. R. China

**Keywords:** chronic disease, gene delivery vectors, mechanosensitive channels, sonogenetics

## Abstract

Sonogenetics is an innovative technology that integrates ultrasound with genetic editing to precisely modulate cellular activities in a non‐invasive manner. This method entails introducing and activating mechanosensitive channels on the cell membrane of specific cells using gene delivery vectors. When exposed to ultrasound, these channels can be manipulated to open or close, thereby impacting cellular functions. Sonogenetics is currently being used extensively in the treatment of various chronic diseases, including Parkinson's disease, vision restoration, and cancer therapy. This paper provides a comprehensive review of key components of sonogenetics and focuses on evaluating its prospects and potential challenges in the treatment of chronic disease.

## Graphical abstract

This article reviews the research progress of sonogenetics in the past decade. As a technology that combines ultrasound and gene editing, sonogenetics non‐invasively regulates target channels in the form of mechanical waves, further affecting cell activity. This has attracted widespread attention in chronic disease treatment such as Parkinson's disease, vision restoration, and cancer therapy. At the same time, this review also provides insights into the potential challenges that sonogenetics may encounter and its future development.

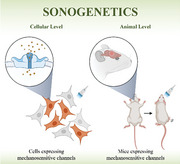

## 1 Introduction

Ultrasound technology is a non‐invasive and safe imaging modality routinely employed in the medical field, playing a crucial role in the detection and diagnosis of various diseases.^[^ ^1^ ^]^ Additionally, ultrasound not only aids in diagnostics but also provides real‐time guidance during surgical procedures.^[^ ^2^ ^]^ Its applications extend beyond diagnosis, encompassing therapeutic interventions. One notable example is high‐intensity focused ultrasound technology, which utilizes specialized generators to produce high‐frequency sound waves focused on a specific point via a transducer.^[^ ^3^ ^]^ This non‐invasive technique treats tumors and pathological tissues by generating a region of high‐intensity energy at the focal point. The fusion of ultrasound technology and genetic engineering has led to the field of sonogenetics, where researchers explore using ultrasound's mechanical energy properties to control gene expression in cells.^[^ ^4^ ^]^ This manipulation of gene expression facilitates the regulation of cell behavior, highlighting the promising applications of ultrasound in the field of sonogenetics.

Sonogenetics, inspired by optogenetics which utilizes light energy to manipulate gene expression within living organisms emerges as an extension of this concept.^[^ ^5^ ^]^ In contrast, sonogenetics employs ultrasonic waves to achieve precise and non‐invasive control over gene regulation. Building upon the success of optogenetics, sonogenetics demonstrates how ultrasonic waves can effectively modulate gene expression, offering a promising approach to non‐invasively manipulate genetics.^[^ ^6^ ^]^ The concept of using ultrasound to stimulate specific genetically labeled cell populations was first proposed by Sreekanth et al of The Salk Institute for Biological Studies, who introduced the term “sonogenetics” to describe this innovative technique.^[^ ^7^, ^8^ ^]^

In sonogenetics, the mechanisms by which ultrasound exerts its effects primarily include mechanical, thermal, and cavitation effects. The mechanical effect directly influences cell membranes and mechanosensitive ion channels by generating mechanical force or stress, altering their state of openness.^[^ ^9^ ^]^ The thermal effect converts ultrasound energy into heat energy, resulting in a localized increase in temperature that activates ion channels.^[^ ^10^ ^]^ The cavitation effect involves the formation and collapse of bubbles, producing high temperatures, high pressures, and shock waves that temporarily rupture cell membranes, thereby increasing the efficiency of gene delivery.^[^ ^7^ ^]^

Cell regulation through sonogenetics employs various mechanisms and methods. For example, sonothermogenetics combines thermal stimulation and ultrasound, utilizing ultrasound‐induced local tissue temperature changes to activate thermosensitive channels.^[^ ^10^ ^]^ Additionally, there are two primary approaches to ultrasound regulation of cellular functions: microbubble‐mediated and non‐microbubble‐mediated sonogenetics. Microbubbles serve as agents that enhance the energy of ultrasound waves.^[^ ^7^ ^]^ Localized mechanical forces generated by rupture of microbubbles can open cell membrane and facilitate genes delivery, however, this process may also result in local tissue damage or inflammatory responses. In contrast, non‐microbubble‐mediated sonogenetics directly uses ultrasound to act on cells, altering the tension of the cell membrane through the mechanical force, thereby triggering the opening of mechanosensitive channels and the transduction of intracellular signals.^[^ ^9^ ^]^

Despite the unique roles of these effects and techniques, the mechanical effect remains the most common and principal mechanism in sonogenetics. Ultrasonic wave, as a mechanical wave, have the unique ability to penetrate intact bone and deep tissue non‐invasively, focusing on small volumes of a few cubic millimeters.^[^ ^11^, ^12^, ^13^, ^14^ ^]^ Sonogenetics involves using a specialized sound wave generator to produce high‐frequency acoustic waves, typically ranging from 20 kilohertz to 1 megahertz.^[^ ^15^ ^]^ These sound waves are focused onto a small focal point through a lens or transducer, with a diameter that can be as small as a few millimeters.^[^ ^16^ ^]^ At this focal point, the energy density of the sound waves increases significantly, exerting a high‐intensity mechanical force. Mechanosensitive channels, acting as mechanosensitive nanovalves and responsive to ultrasound stimulation, can be affected by the vibrational action of the sound waves through genetic technology.^[^ ^6^, ^17^, ^18^ ^]^ This mechanical effect alters the permeability of the cell membrane, leading to the opening and closing of ion channels and, consequently, modifying intracellular signal transduction pathways.^[^ ^19^ ^]^ Ultimately, this process modulates the functionality of the target cells.

Importantly, this non‐invasive mode of regulation enhances patient comfort and compliance. Additionally, the controllability and reversibility of sonogenetics offer more flexible options for personalized treatment. Consequently, sonogenetics holds great potential in the management of various chronic disease. For instance, neurological disorders affecting specific spatial regions of neural circuits are common in the adult population.^[^ ^20^, ^21^ ^]^ Understanding brain functions and addressing conditions such as depression, anxiety, Parkinson's disease, and migraines necessitates the precise definition and manipulation of specific neurons.^[^ ^22^, ^23^, ^24^ ^]^ Various neuromodulation techniques, including optogenetics, electrical, magnetic, and chemogenetics, have been developed for this purpose.^[^ ^25^, ^26^, ^27^, ^28^, ^29^ ^]^ Optogenetics enables highly precise control and rapid response, but its application is limited in deeper tissues due to the restricted penetration of light. Magnetogenetics, on the other hand, allows for non‐invasive remote regulation of deep‐brain neurons using magnetic fields, but the complexity of magnetic equipment and the biocompatibility concerns associated with magnetic particles remain challenges. Additionally, its spatial and temporal precision is relatively lower.^[^ ^30^, ^31^, ^32^ ^]^ In short, although these methods have provided valuable insights into cell manipulation in different model systems, they have limitations such as invasiveness, poor tissue penetrability, limited utility in deeper tissue target cells, and low spatiotemporal precision.^[^ ^15^, ^33^, ^34^ ^]^ To address these limitations, sonogenetics provides a non‐invasive alternative with excellent penetrability and high spatiotemporal precision. Building on this research, akin to treatment strategies used in neurological disorders, studies have shown that sonogenetics can induce discharge activity not only in neurological cells. These discoveries present exciting possibilities for significant medical applications. For instance, precise control of cellular activity in the retina shows potential for developing therapies to address visual impairments or degenerative eye conditions. Likewise, the modulation of cardiac cells through sonogenetics could advance in cardiac pacing or arrhythmia management. The ability to elicit of limb movements creates opportunities for therapeutic interventions in conditions affecting motor functions. Overall, the expanding applications of sonogenetics not only enhances our understanding of cellular responses but also indicate its potential in addressing a wide range of medical challenges. Further exploration of these applications is crucial to full unlock the therapeutic potential of sonogenetics in various medicine fields.^[^ ^35^, ^36^, ^37^, ^38^, ^39^ ^]^

Previous reviews on sonogenetics have primarily focused on the research and applications of this field in monitoring and regulating biomolecular functions, fundamental principles and mechanisms, broad applications in cellular regulation, as well as neuroregulation and therapeutic potential.^[^ ^8^, ^40^, ^41^, ^42^, ^43^ ^]^ This review focuses on the application of sonogenetics in the treatment of chronic diseases. It particularly summarizes the latest research advances in mechanosensitive channels and gene delivery systems, and thoroughly discusses their applications, potential challenges, and future prospects in the treatment of chronic diseases.

## 2 The Sonogenetics Toolkit

Mechanosensitive channels are integral components of various organs and tissues, playing critical roles in sensing external mechanical stimulation and internal pressure changes.^[^ ^44^ ^]^ In neurons, mechanosensitive channels such as MEC‐4 and calcium‐selective mechanosensitive ion channels naturally exist. These channels can respond to ultrasound stimulation, leading to calcium ion accumulation and the generation of action potentials.^[^ ^6^, ^45^ ^]^ In auditory cortical neurons, low‐intensity of ultrasound can elicit direct action potentials.^[^ ^46^ ^]^ Retinal neurons exhibit responses to ultrasound stimulation, suggesting that mechanosensitive channels may naturally reside in retinal neurons.^[^ ^47^ ^]^

For organs or tissues lacking mechanosensitive channels, the introduction of these channels can enhance the perception of mechanical stimulation, thereby helping to restore or improve their function.^[^ ^15^ ^]^ With the advancement of gene delivery technologies, researchers are exploring the integration of mechanosensitive channels into organs or tissues that naturally lack them, which holds the promise of providing precise, non‐invasive control of cellular functions. The tools required for implementing sonogenetics primarily include mechanosensitive channels, gene delivery vectors, and ultrasound stimulation devices.^[^ ^48^ ^]^ Ultrasound is a mechanical wave with mechanical effects.^[^ ^49^ ^]^ Mechanosensitive channels are ion channels that facilitate cellular responses to mechanical force stimulation, capable of converting mechanical force into electrical and chemical signals.^[^ ^50^ ^]^ Electrical signals are typically generated through the transmembrane movement of ions such as Ca^2^⁺, Na⁺, and K⁺, leading to changes in membrane potential.^[^ ^51^ ^]^ Chemical signals involve intracellular messenger molecules, such as Ca^2^⁺ ions as a secondary messenger, triggering cascade reactions that regulate gene expression, protein synthesis, and other cellular functions.^[^ ^52^ ^]^

These channels can be introduced into target cells using viral or non‐viral vectors and expressed on the target cell membrane.^[^ ^17^ ^]^ When subjected to appropriate ultrasound stimulation, these channels can transition between open and closed states, thereby modulating ion permeability and influencing intracellular signal transduction and overall cellular function.^[^ ^18^, ^53^ ^]^

## 3 Mechanosensitive Channels of Sonogenetics

**Figure 1:**
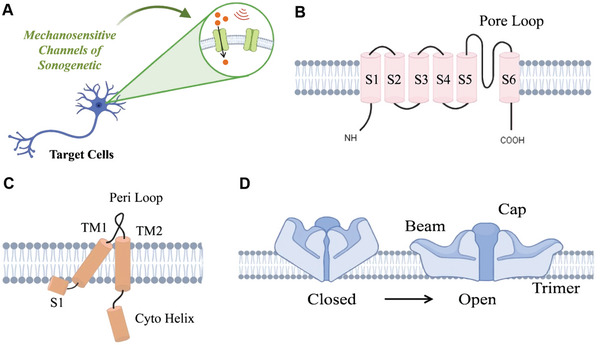
Mechanosensitive channels commonly used in sonogenetics. A) Ion transmembrane movement occurs in mechanosensitive channels under ultrasound stimulation. B) TRP is composed of four identical or similar subunits, each consisting of six transmembrane segments with cationic channels between segments 5 and 6.^[^ ^59^ ^]^ C) MscL is a homopentamer, with 136 amino acid subunits each forming two transmembrane α‐helices (TM1 and TM2) linked by a periplasmic loop.^[^ ^60^ ^]^ D) Piezo 1 exhibits a unique propeller like three leaf homomorphic shape, each containing a peripheral blade, an anchor, and a beam on the inner side of the cell.^[^ ^61^ ^]^ Created with BioRender.com.

Mechanosensitive channels are pore‐forming membrane proteins that facilitate the passage of ions through their channel pores under mechanical strain including light, ultrasound, magnetism, etc.^[^ ^17^, ^54^, ^55^ ^]^ These channels play a crucial role in regulating ion concentrations both inside and outside of cells, acting as molecular switches to control cell activity (**Figure** **1**).^[^ ^56^ ^]^ Ultrasound, a mechanical stimulus, can activate mechanosensitive channels embedded within cellular membranes.^[^ ^57^, ^58^ ^]^ To be effective, these channels must meet specific criteria: a) have high sensitivity to ultrasound stimulation; b) expression in mammalian cells; c) no alteration of electrical properties or cell survival post‐expression. Various mechanosensitive ion channels like Transient receptor potential (TRP), large conductance mechanosensitive channel protein (MscL), Piezo1, Prestin, two‐pore‐domain potassium family, and MEC‐4/6, have been implicated in cellular responses to ultrasound. Meanwhile, mutants of mechanosensitive channels have demonstrated more enhanced sensitivity and more precise regulation of cellular in response to ultrasound stimulation (**Table** **1**).

**Table 1:** Key mutations or family members of mechanosensitive channels.

Mechanosensitive channels	Mutations/ family members	Mutational site	Characteristic	Ref.
TRP	TRP‐4	/	TRP‐4 is expressed in the nervous system of *Caenorhabditis elegans*.	[7]
	hsTRPA1	/	TRPA1 is the only member of the mammalian family.	[62]
	TRPV1	/	TRPV1 is affected by both thermal and mechanical effects.	[63]
MscL	MscL I92L	Isoleucine at position 92 was substituted with Leucine	I92L mutation can increase the sensitivity of MscL to ultrasound, and can be activated at peak negative pressures as low as 0.25 MPa, in the absence of microbubbles. Meanwhile, I92L mutant has millisecond temporal precision.	[17]
	MscL‐G22S	Bearing a glycine to serine substitution at position 22	G22S mutation has a lower threshold for gating than wild‐type MscL but does not show spontaneous activity.	[64]
Piezo	Piezo1	/	Piezo is the most sensitive mechanotransduction ion channel.	[65]
Prestin	mPrestin (N7T, N308S)	Asn at positions 7 and 308 is replaced with Thr and Ser	Compared with WT Prestin, mPrestin (N7T, N308S) is more sensitive under low‐frequency (0.5 MHz), low‐energy (0.5 MPa and 0.1% of duty cycle), and transient (3 s) US conditions.	[66]

### 3.1 Transient Receptor Potential (TRP) Channels in Sonogenetics

**Figure 2:**
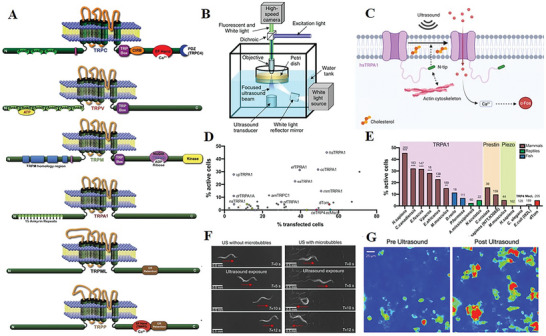
TRP family members respond to ultrasound and trigger cellular and animal responses. A) Molecular domains of TRP channels.^[^ ^59^ ^]^ B) The structure of ultrasound exposure system.^[^ ^7^ ^]^ C) Schematic diagram of TRP activation process in cells.^[^ ^62^ ^]^ D,E) Activation percent of mechanosensitive‐protein‐transfected cells after ultrasound stimulation.^[^ ^62^ ^]^ F,G) The response of cells and nematodes expressing TRP after ultrasound stimulation.^[^ ^7^, ^73^ ^]^ Reproduced with permission.^[^ ^59^ ^]^ Copyright 2010, American Society for Pharmacology and Experimental Therapeutics. Reproduced with permission.^[^ ^7^ ^]^ Copyright 2015, Springer Nature. Reproduced with permission.^[^ ^62^ ^]^ Copyright 2022, Springer Nature. Reproduced with permission.^[^ ^73^ ^]^ Copyright 2021, CSH.

TRP was initially identified in 1969 as a response to photoexcitation in a mutant of Drosophila melanogaster mutant. Subsequently, TRP channels have been discovered in almost all eukaryotes, making them potential candidates for mechanotransductive channels (**Figure** **2**A).^[^ ^59^, ^67^, ^68^, ^69^ ^]^ TRP channels are involved in a range of sensory responses such as heat, cold, pain, pressure, vision, and taste. Additionally, they play a crucial role in the regulation of intracellular Ca^2+^ concentration.^[^ ^70^, ^71^, ^72^ ^]^

Since 2015, TRP has been used as an ultrasound sensitive ion channel. Chalasani et al. discovered that Caenorhabditis elegans with misexpressed TRP‐4 exhibit heightened sensitivity to low‐pressure ultrasound compared to wild‐type organisms (Figure 2F). This enabled the successful manipulation of interneurons and sensory neurons expressing TRP‐4 using low‐pressure ultrasound stimulation.^[^ ^7^ ^]^ However, the use of low‐frequency ultrasound may result in limited spatial resolution and unfocused effect. Therefore, in 2022, a 7 MHz frequency was chosen for stimulation. To identify the most suitable mechanosensitive channel for sonogenetics, 191 candidate proteins were transfected into HEK293 cells. It was determined that cells expressing hsTRPA1 showed the most promising response to ultrasound stimulation, effectively allowing for the manipulation of neurons within the intact mammalian brain (Figure 2D,E).^[^ ^62^ ^]^ Friend et al also selected hsTRPA1 as a mechanosensitive channel further investigation in the same year. By transfecting hsTRPA1 into HEK cells and utilizing transducer‐mounted diffusers for even distribution of ultrasound in irregular cavities, an increase in calcium uptake and activation of a larger number of cells in hsTRPA1 neurons was observed (Figure 2B,C,G).^[^ ^73^ ^]^

Chen et al. conducted a study to assess the feasibility and safety of TRPV1‐mediated. They transfected TRPV1 into the motor cortex of freely moving mice in order to modulate their locomotor behavior. The researchers observed an increase in c‐Fos, indicating cortical neuron activation, as well as alterations in inflammatory and apoptotic markers to assess potential thermal‐induced tissue damage. The experimental data suggested that ultrasound stimulation at 0.7 MPa effectively activated cortical neurons and evoked rotational behavior in the opposite direction of the stimulation site with no significant changes in markers. However, ultrasound stimulation at 1.1 MPa caused damage to the meninges.^[^ ^63^ ^]^

Numerous studies have demonstrated a strong correlation between the structure and function of TRP, and functional amino acid mutations such as TRPV1‐S512Y and TRPV4‐R316C can affect responsiveness of TRP to thermal, electrical, and mechanical stimuli.^[^ ^74^, ^75^, ^76^ ^]^ The investigation of these mutants aids in the identification of mechanosensitive channels that are more suitable for clinical application conditions, providing new opportunities for the clinical translation of sonogenetics.

### 3.2 Large Conductance Mechanosensitive Channel Protein (MscL) in Sonogenetics

**Figure 3:**
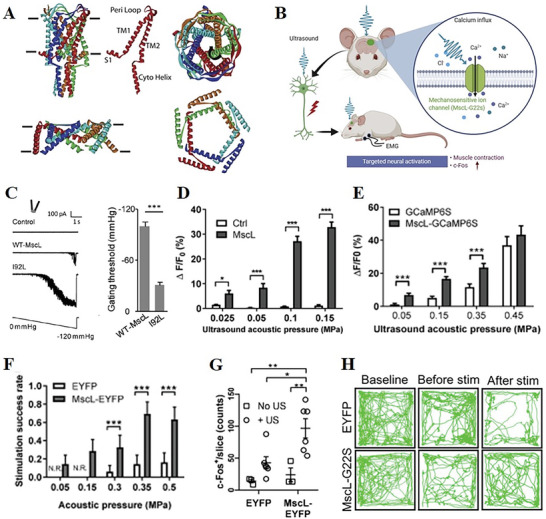
MscL channel and its mutants respond to ultrasound and trigger cellular and animal responses. A) Molecular domains of MscL channel and models of open and closed states.^[^ ^60^ ^]^ B) Schematic diagram of MscL activation process in cells.^[^ ^64^ ^]^ C) Representative currents and gating thresholds of WT‐ or I92L‐MscL‐infected cells.^[^ ^17^ ^]^ D–G) Cells expressing MscL showed increased Ca^2+^ influx, greater muscular responses, more active neurons, and more expressions of c‐Fos in response to ultrasound.^[^ ^64^ ^]^ H) Ultrasound stimulation improved motor coordination behavior of PD mice.^[^ ^87^ ^]^ Reproduced with permission.^[^ ^60^ ^]^ Copyright 2012, American Society for Microbiology. Reproduced with permission.^[^ ^64^ ^]^ Copyright 2020, Elsevier. Reproduced with permission.^[^ ^17^ ^]^ Copyright 2018, American Chemical Society. Reproduced with permission.^[^ ^87^ ^]^ Copyright 2023, PNAS.

MscL is extensively examined in terms of biophysics and structures. Originating from Escherichia coli, MscL is composed of a homopentamer, with each subunit consisting of 136 amino acids that form two transmembrane α‐helices (TM1 and TM2) connected by a periplasmic loop (**Figure** **3**A). This protein has the ability to detect changes in membrane tension (Figure 3C).^[^ ^60^, ^77^, ^78^, ^79^, ^80^ ^]^ When tension is applied to the lipid bilayer, MscL acts as a force‐from‐lipid channel, causing deformation in the internal force profile of the lipid bilayer. This deformation leads to the movement of helices, resulting in the opening of a pore ≈30 Å in diameter.^[^ ^81^, ^82^, ^83^ ^]^ Ions and molecules with a molecular weight of less than ≈1000 are able to pass through without being selectively filtered.^[^ ^84^, ^85^ ^]^

In 2018, Jia et al conducted a study where they expressed MscL in cultured rat hippocampal neurons and demonstrated its activation through low‐pressure ultrasound. They also discovered that the I92L mutation, a gain‐of‐function mutation, increased the sensitivity of MscL to acoustic stimulation through TM1‐TM2 interaction. The I92L mutant MscL exhibited higher sensitivity compared to the wild‐type MscL, resulting in a lower activation threshold of −31±10 mm Hg and the ability to generate a series of precisely timed spikes.^[^ ^17^ ^]^ In 2020, Chang et al designed a logic AND‐gated nanosystem for regulating tumor cell apoptosis. The gate, MscL I92L, could be opened by ultrasound, allowing Ca^2+^ influx to activate the cell apoptosis pathway.^[^ ^86^ ^]^

In addition to the I92L mutant, different mutations with different sensitivities to stimulation have been studied. In 2020, Sun et al first introduced MscL‐G22S to sensitize 293T cells to ultrasound (Figure 3B). By progressively expressing MscL‐G22S into cells, they observed consistent ultrasound‐induced Ca^2+^ influx and increased neuronal activation at lower intensities in excitatory neurons in the cortices of mice and the right dorsomedial striatum (Figure 3C–G).^[^ ^64^ ^]^ Subsequently, in 2023, it was demonstrated that ultrasound can activate defined neural pathway to alleviate the symptoms of neurodegenerative diseases (Figure 3H).^[^ ^87^ ^]^

Previous research has shown that high‐frequency ultrasound can have an inhibitory effect on neural modulation and can cause thermal damage to tissue. Therefore, low‐frequency ultrasound is commonly employed for neuronal activity modulation. However, this approach often results in prolonged responses and limited spatial resolution. To improve spatiotemporal resolution, Serge et al used high‐frequence ultrasound at low acoustic intensities to activate MscL with G22S mutant targeted in rat retinal ganglion cells. Their research confirmed that sonogenetics can provide millisecond pattern presentations.^[^ ^16^ ^]^

### 3.3 Piezo 1 Channels in Sonogenetics

**Figure 4:**
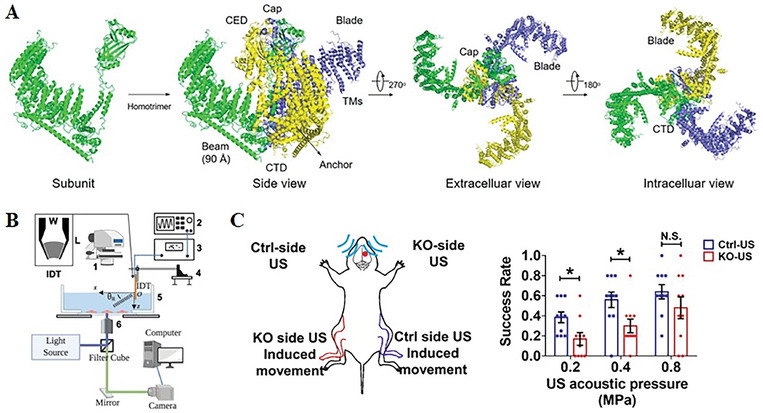
Piezo channel responds to ultrasound and trigger animal responses at varying acoustic pressures. A) Cryo‐EM structure of mouse Piezo1 protein.^[^ ^61^ ^]^ B) Schematic illustration of system used to monitor concomitantly the response of cells to ultrasound stimulation.^[^ ^19^ ^]^ C) The effect of ultrasound stimulation on mouse hind limb movement.^[^ ^65^ ^]^ Reproduced with permission.^[^ ^61^ ^]^ Copyright 2022, American Chemical Society. Reproduced with permission.^[^ ^19^ ^]^ Copyright 2021, Springer Nature. Reproduced with permission.^[^ ^65^ ^]^ Copyright 2023, PNAS.

Piezo 1 is a eukaryotic mechanosensitive ion channel that specifically responds to mechanical stimulation induced by ultrasound.^[^ ^88^, ^89^ ^]^ In contrast to other mechanosensitive ion channels, Piezo1 exhibits a diverse expression pattern in mammalian cells, high specificity and sensitivity to mechanical force. It also demonstrates a frequency‐dependent filtering effect in response to repetitive forces.^[^ ^90^, ^91^, ^92^, ^93^ ^]^ Comprised of three identical proteins, each containing 2500 amino acid residues, Piezo 1 is a substantial structure.^[^ ^94^, ^95^, ^96^ ^]^ Structural and functional studies have revealed that Piezo 1 undergoes conformational changes when mechanically stimulated, influenced by both lateral membrane tension (force‐from‐lipid) and central filament tension (force‐from‐silk), enabling for selective transmembrane transport of cations (**Figure** **4**A).^[^ ^61^, ^97^ ^]^

Multiple studies have explored the impact of ultrasound on Piezo1. In 2021, Zhong et al investigated the effect of different ultrasound exposure conditions on Piezo 1 activation and intracellular calcium response. The study revealed that the shear stress amplitude and pulse length of the stimulation were the primary factors influencing channel activation and Ca^2+^ influx. Under the same insonification energy and treatment time (Figure 4B), they set the value of pulse length to 20 ms, and found that constant shear stress of 50 dyne cm^2^ could achieve the highest Piezo1 activation probability and the strongest Ca^2+^ influx, under the premise of ensuring cell detachment or membrane injury < 10%.^[^ ^19^ ^]^ In contrast, Sun et al. selectively knocked out Piezo1 in targeted cells instead of expressing it in HEK293T cells. They discovered that Piezo1 expression in the central amygdala (CEA) exceeded that in the motor cortex of mice, making CEA more sensitive to ultrasound. Furthermore, the results of the study suggest that Piezo 1 may regulate neural activity in response to ultrasound, as evidenced by markedly reduced neuronal calcium responses, limb movement, and muscle electromyogram responses observed in both the CEA and motor cortex following Piezo 1 knockout (Figure 4C).^[^ ^65^ ^]^

Similarly, mutations in the Pizeo1 protein are associated with various diseases, particularly blood and bone disorders. Genetic diseases such as Piezo1‐R2456H and Piezo1‐M2225R mutants are associated with sickle cell anemia, affecting the homeostasis of red blood cell volume.^[^ ^98^ ^]^

### 3.4 Prestin as a Piezoelectric Amplifier in Sonogenetics

**Figure 5:**
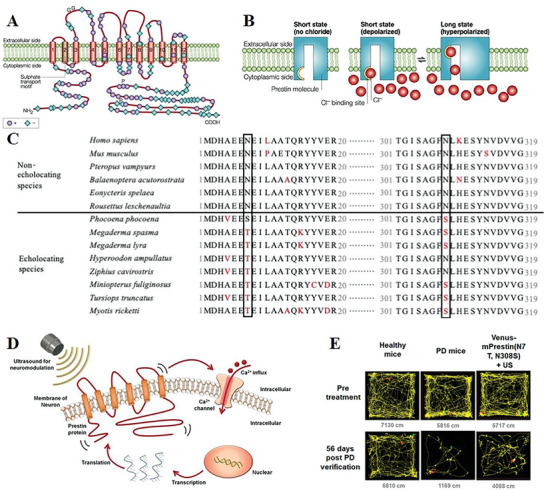
Prestin protein responds to ultrasound and trigger cellular and animal responses. A) Molecular domains of Prestin protein.^[^ ^99^ ^]^ B,D) Schematic diagram of Prestin activation process in cells.^[^ ^99^, ^104^ ^]^ C) N7T and N308S substitutions in the echolocating species.^[^ ^105^ ^]^ E) Ultrasound stimulation improved motor function recovery of PD mice.^[^ ^4^ ^]^ Reproduced with permission.^[^ ^99^ ^]^ Copyright 2002, Springer Nature. Reproduced with permission.^[^ ^104^ ^]^ Copyright 2020, Ivy spring Int Publ. Reproduced with permission.^[^ ^105^ ^]^ Copyright 2019, American Chemical Society. Reproduced with permission.^[^ ^4^ ^]^ Copyright 2021, American Chemical Society.

Prestin, unlike other mechanosensitive proteins in sonogenetics, functions as a piezoelectric amplifier rather than a pore‐forming channel (**Figure** **5**A,B,D).^[^ ^99^ ^]^ This transmembrane protein, found in the mammalian auditory system, acts as an electromechanical transducer.^[^ ^100^ ^]^ While Prestin in human shows minimal sensitivity to ultrasound stimulation, it exhibits high sensitivity to high‐frequency sound waves in echolocating mammals like dolphins, cetaceans, and bats.^[^ ^101^, ^102^ ^]^ Lin et al analyzed Prestin's amino acid sequences, noting differences between echolocating and non‐echolocating species. Specifically, they observed that echolocating species frequently replace Asn at positions 7 and 308 with Thr and Ser (Figure 5C). Introducing mutations N7T and N308S into Prestin and transfecting it into human HEK293T cells, they found that the mPrestin(N7T, N308S) group had a higher percentage of cells responsive to 0.5 MHz focused ultrasound compared to the wild‐type group.^[^ ^103^ ^]^ In 2020, Yeh et al used a non‐invasive gene delivery method for Prestin and observed increased c‐Fos staining in cells expressing Prestin.^[^ ^104^ ^]^ Next year, mPrestin(N7T, N308S) was expressed in the dopaminergic neurons of Parkinson's disease mice in the substantia nigra. The experiment and analysis demonstrated that repeated and localized stimulation using 0.5 MHz US can ameliorate the dopaminergic neurodegeneration and mitigate the PD symptoms when compared to control group (Figure 5E).^[^ ^4^ ^]^

### 3.5 Other Mechanosensitive Channels in Sonogenetics

In addition to mechanosensitive proteins mentioned above, various ion channels, including TRAAK, have been implicated in sonogenetics. TRAAK, a K^+^ channel expressed in neurons and retinal cells that plays a significant role in physiological functions such as neuronal discharge, heart pumping, and insulin secretion. Stephen et al highlighted that membrane tension is pivotal for TRAAK activation, as it exhibits minimal open probability in the absence of such tension.^[^ ^106^ ^]^ Similar, Meng et al. studied the impact of ultrasound stimulation on MEC‐4 and MEC‐6 ion channels using Caenorhabditis elegans behavioral responses. Mutant worms showed reduced reversal behavior (30% ± 10.5% and 10% ± 6.9%, respectively) compared to wild type (85% ± 8.2%), suggesting the involvement of MEC‐4 and MEC‐6 in the response of Caenorhabditis elegans to ultrasound stimulation.^[^ ^107^ ^]^

## 4 Gene Delivery Systems of Sonogenetics

**Figure 6:**
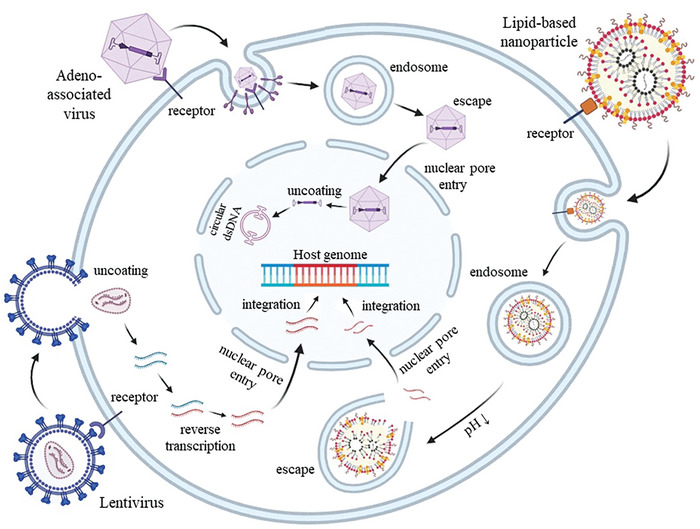
Gene delivery systems of sonogenetics. In sonogenetics researches, two main types of gene delivery systems are usually used: virus based delivery systems (AAV, LV) and non‐virus based delivery systems (LNP). The ways in which carriers deliver genes within the cell are shown in this figure. Created with BioRender.com.

The mechanical sensitive channel was previously discussed, and the next critical step in sonogenetics involves expressing mechanosensitive channel proteins on target cells. Gene delivery systems play a vital role in this process by loading target genes into vectors and delivering them to target cells for expression.^[^ ^108^ ^]^ In the current researches, two main types of gene delivery systems are used: virus based delivery systems and non‐virus based delivery systems. Here, we will provide a detailed introduction to the three most commonly used methods: adeno‐associated virus (AAV), lentivirus (LV), and lipid‐based nanoparticle (LNP) (**Figure** **6**). Viral vectors, such as AAV and LV, are highly efficient in delivering genes to target cells with sustained and stable gene expression, which is particularly beneficial for long‐term applications such as chronic disease treatment. AAV, for example, provides prolonged gene expression with minimal immunogenicity, making it a popular choice in sonogenetics. However, its limited cargo capacity and potential integration into the host genome present risks of insertional mutagenesis. Non‐viral vectors, such as LNPs, offer higher safety profiles due to their lack of immunogenicity and do not carry the risk of genomic integration. However, they generally exhibit lower transfection efficiency and shorter durations of gene expression compared to viral vectors, making them more suitable for temporary or short‐term therapeutic interventions. Additionally, non‐viral vectors often require repeated administrations to maintain therapeutic effects, which can be a disadvantage in applications where long‐term gene expression is critical.

### 4.1 Virus Based Delivery Systems: Biological Methods

Viruses are a type of microorganisms that can effectively infect human cells and other animal cells.^[^ ^109^ ^]^ Upon infecting a host cell, a virus inserts its genetic material into the cell's DNA, aiding in the production of proteins and the assembly of new virus particles.^[^ ^110^, ^111^ ^]^ Researchers propose modifying natural viruses by incorporating specific gene fragments into the virus genome sequence, utilizing the virus as a carrier for targeted gene delivery.^[^ ^112^ ^]^ This approach takes advantage of the virus's specific process of infection in host cells and the protective protein coat that shields its internal genetic material from degradation by host enzymes. In the 1990s, researchers successfully utilized retroviruses and adenosine deaminase genes to restore the basic health of patients with severe combined immunodeficiency syndrome.^[^ ^113^, ^114^ ^]^ Currently, various virus delivery systems are still prevalent in clinical trials and even disease treatment.^[^ ^115^ ^]^ It is worth noting that the vast majority of approved gene therapy products in the European and American markets are based on viral vectors.^[^ ^114^ ^]^

#### 4.1.1 AAV

**Figure 7:**
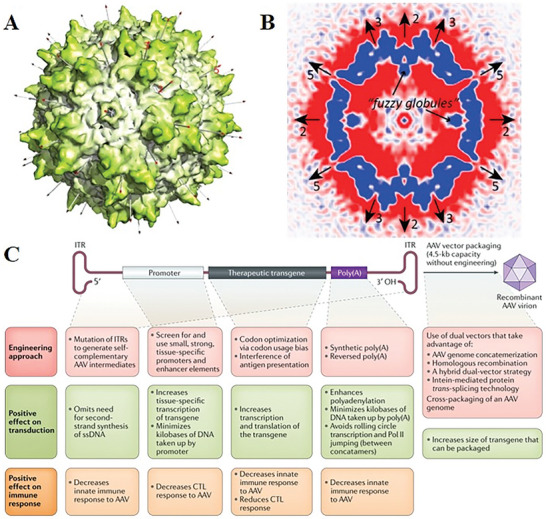
AAV is an unencapsulated icosahedral virus with a diameter of 25 nm, whose genetic material consists of a single‐stranded DNA genome 4.7 kb in length. A) Capsid structure of AAV.^[^ ^117^ ^]^ B) Cryo‐EM reconstruction of AAV empty particles at 1 nm resolution.^[^ ^117^ ^]^ C) AAV cassette and parts that can be engineered.^[^ ^131^ ^]^ Reproduced with permission.^[^ ^117^ ^]^ Copyright 2022, American Chemical Society. Reproduced with permission.^[^ ^131^ ^]^ Copyright 2020, Springer Nature.

AAV is an unencapsulated icosahedral virus with a diameter of ≈25 nm (**Figure** **7**A,B). Its genetic material consists of a single‐stranded DNA genome ≈4.7 kb in length.^[^ ^116^, ^117^ ^]^ AAV is widely utilized in gene therapy as a vector.^[^ ^118^, ^119^ ^]^ Upon entry into the human body, AAV can attach to cell surface receptors, internalize, and move to the nucleus.^[^ ^120^, ^121^ ^]^ Inside the nucleus, AAV sheds its shell, releasing single‐stranded DNA and gene fragments for transcription and translation outside the chromosome, contributing to subsequent genome editing processes.^[^ ^122^, ^123^, ^124^, ^125^ ^]^ However, the presence of AAV‐introduced genes in a free state increases the risk of loss during cell division and the possibility of insertional mutagenesis still exists, despite the reduced risk of integration with host genes.^[^ ^125^, ^126^, ^127^, ^128^ ^]^ Additionally, the immunogenicity stemming from the capsid protein of AAV could significantly impact therapeutic outcomes.^[^ ^129^, ^130^ ^]^ Moreover, due to its small size, AAV can only accommodate a load sequence of ≈4.7 kb, limiting its application in certain cases (Figure 7C).^[^ ^131^, ^132^ ^]^

Up to now, AAV mediated gene delivery systems have been effectively utilized in animal models to treat various diseases, such as ocular disorders, CNS disorders and hemophilia.^[^ ^133^, ^134^, ^135^ ^]^ Building upon the success of numerous animal studies, the first clinical trial based on AAV vector delivery gene editor was conducted in 2012, successfully inserting the correct enzyme human lipoprotein lipase gene into the patients with lipoprotein lipase deficiency.^[^ ^108^ ^]^

In sonogenetics studies, AAV has been extensively utilized by researchers. In 2019, Lin et al used AAV to deliver the calcium biosensor cyan fluorescence protein and Prestin (N7T, N308S) tagged with the yellow fluorescence protein Venus.^[^ ^103^ ^]^ The expression of MscL‐G22S was verified by qPCR and fluorescence imaging, revealing a 450‐fold increase compared to the control group in a mock transfection control. Subsequently, genes encoding mechanosensitive proteins like MscL‐G22S and TRPV1, were transported using AAV, along with markers such as enhanced yellow fluorescence protein (EYFP), Rhodamine dye or red fluorescence protein dTomato (dTom) to detect the transfection status of the target gene fragment.^[^ ^4^, ^16^, ^64^, ^87^ ^]^ For example, Marc et al transiently transfected hsTRPA1 along with dTom, and over 50% of the cells per field of view imaged were positive for dTom.^[^ ^62^ ^]^ Also using dTom, Sara et al found that 24% and 46% of cells expressing dTom for the WT MscL and G22S MscL proteins, and 33.4% of cortical neurons in the transfected area expressed dTom. These data indicated that the transfection effect of AAV in vivo and in vitro is significant.^[^ ^16^ ^]^

#### 4.1.2 LV

**Figure 8:**
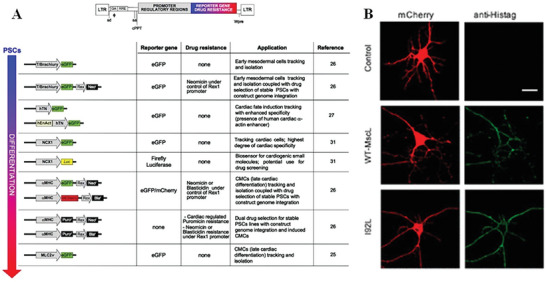
LV contains two 9 kb positive single‐stranded RNA genomes, which can then be integrated into the host chromosome for long‐term stable expression. A) Schematic representation of various LV construct.^[^ ^137^ ^]^ B) The responsiveness of cells transfected through LV to ultrasound stimulation.^[^ ^17^ ^]^ Reproduced with permission.^[^ ^137^ ^]^ Copyright 2012, Springer Nature. Reproduced with permission.^[^ ^17^ ^]^ Copyright 2018, American Chemical Society.

As a member of the retrovirus family, LV is an envelope virus measuring 80–120 nm in diameter. It contains two 9kb positive single‐stranded RNA genomes.^[^ ^136^, ^137^ ^]^ Once inside the cell, LV undergoes reverse transcription to form double‐stranded DNA, which can then be integrated into the host chromosome for long‐term stable expression.^[^ ^138^, ^139^ ^]^ LV vectors are primarily utilized in in vitro gene therapy due to their wide infection range and a carrying capacity of up to 10 kb, allowing for a diverse range of applicable diseases (**Figure** **8**A).^[^ ^140^ ^]^

While lentiviral vectors offer numerous advantages as a delivery system, their random integration and persistent gene expression can also pose risks. The random integration of viral DNA may activate oncogenes or inhibit tumor suppressor genes, potentially leading to tumorigenesis.^[^ ^141^, ^142^, ^143^ ^]^ In addition, stable gene editor expression may result in unintended cleavage at sites with similar sequences to the target, causing off‐target cleavage throughout the genome and potentially harmful mutations.^[^ ^144^ ^]^ Despite these concerns, LV still demonstrates positive therapeutic effects in the treatment of severe combined immunodeficiency and β‐thalassemia, with no reported side effects at present.^[^ ^124^, ^145^, ^146^ ^]^

In the field of sonogenetics, Ye et al transfected histidine‐tagged MscL into rat hippocampal neurons in a primary culture through LV and tested the transfection status through immunostaining (Figure 8B).^[^ ^17^ ^]^ The James Friend research group used fluorescence staining to detect transfection, using dTom as a fluorescence reporter.^[^ ^73^ ^]^ Compared to non‐viral vectors, viral vectors have the advantages of high targeting and high transduction efficiency.^[^ ^115^ ^]^ However, it is crucial to consider that viral vectors may induce some level of immunogenicity, potentially eliciting a robust immune response.^[^ ^147^, ^148^ ^]^ Moreover, certain viral vectors have the capability to integrate their own genetic material into the host cell's genomic DNA, posing a risk of compromising the integrity of the host genome.^[^ ^143^ ^]^

### 4.2 Non‐Virus Based Delivery Systems: Chemical Methods

While viral vectors exhibit superior delivery efficiency and cellular uptake capabilities, they also present certain drawbacks and potential issues. First, viral vectors typically possess high immunogenicity, which can potentially trigger immune responses that may limit their effectiveness.^[^ ^149^ ^]^ Second, viral vectors often have a limited gene payload capacity, restricting their ability to carry and deliver larger gene sequences.^[^ ^150^ ^]^ Third, different types of viral vectors exhibit limited cell‐type selectivity, potentially limiting their broad applicability Applicability.^[^ ^151^ ^]^ Fourth, some viral vectors integrate their genetic material into the host cell's genome, leading to irreversible impacts on the host genome. This raises safety and ethical concerns in the context of gene therapy.^[^ ^152^ ^]^ Finally, the production and preparation of viral vectors often involve intricate engineering and cultivation conditions, demanding high levels of biosafety measures.^[^ ^153^ ^]^ This not only increases production costs but also limits the feasibility of large‐scale manufacturing.

**Figure 9:**
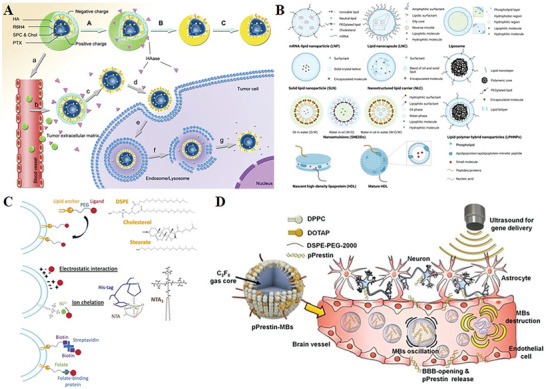
Schematic design of LNP for gene delivery. A) Schematic diagram of the dual‐decorated LNP delivering tumor targeted drugs.^[^ ^156^ ^]^ B) Structures of different types of LNP.^[^ ^158^ ^]^ C) Noncovalent surface modification techniques of LNP.^[^ ^158^ ^]^ D) Ultrasound‐responsive LNPs deliver Prestin plasmid.^[^ ^104^ ^]^ Reproduced with permission.^[^ ^156^ ^]^ Copyright 2022, American Chemical Society. Reproduced with permission.^[^ ^158^ ^]^ Copyright 2012, Elsevier. Reproduced with permission.^[^ ^104^ ^]^ Copyright 2020, Ivyspring Int Publ.

Researchers are currently focusing on improving the safety, controllability, and applicability of viral vectors, as well as exploring alternative non‐virus based delivery systems such as plasmid transfection, electroporation techniques, and liposomes. Liposomes, in particular, have shown promise as a chemical component for delivery.^[^ ^154^ ^]^ The concept involves encapsulating RNA fragments encoding the target protein within hollow liposome nanoparticles (**Figure** **9**A). These liposomes can fuse with human cells upon injection, delivering the RNA fragments and facilitating the translation of the target protein to achieve the desired therapeutic outcome.^[^ ^155^ ^]^ While cationic liposome transfection reagents are commonly used for in vitro cell transfection, their high cytotoxicity and limited transfection efficiency in vivo have raised concerns about their safety and efficacy for in vivo applications.^[^ ^156^, ^157^ ^]^ Consequently, researchers have attempted to modify liposome delivery systems to achieve higher safety and efficiency, which led to the development of LNP.^[^ ^158^ ^]^

LNP is currently used less frequently in sonogenetics research compared to viral vector‐based delivery systems. The Jin Chang research group and Wu Chunyao research group both utilized LNP to transfect green fluorescence protein labeled MscL I92L and pPrestin, respectively, and evaluated the gene transfection rate using fluorescence microscopy (Figure 9D).^[^ ^86^, ^104^ ^]^ Wu et al discovered that gene transfection requires an acoustic pressure exceeding 0.3 MPa, with a pressure of 0.5 MPa striking the optimal balance between enhancing transfection efficiency and maintaining cell viability. They ultimately concluded that under optimal acoustic parameters of 0.5 MPa, 5 Hz of PRF, and 5000 for cycle number, gene transfection rate is 24.2±0.9%.

Compared with viral vectors, non‐viral vectors have a lower transfection rate, but they offer unique advantages. LNP is a complex delivery technology that consists of four key lipid types: ionizable lipids, polyethylene glycol lipids, auxiliary phospholipids, and cholesterol (Figure 9B,C).^[^ ^158^, ^159^ ^]^ Ionizable lipids are pH‐dependent and neutral in serum, facilitating effective cellular uptake. Inside cells, the acidic vesicle environment triggers ionizable lipids to form cations, releasing target RNA fragments. This pH dependence minimizes systemic toxicity.^[^ ^160^, ^161^, ^162^, ^163^ ^]^ PEG lipids enhance particle stability by preventing immune recognition, while auxiliary phospholipids and cholesterol further stabilize LNP structure.^[^ ^164^, ^165^ ^]^ As LNP lacks externally exposed protein or peptide components, it is considered to have lower immunogenicity.^[^ ^166^, ^167^ ^]^ However, due to the composition of various lipid molecules, LNPs tend to bind to apolipoproteins in the bloodstream, leading to accumulation in the liver.^[^ ^168^ ^]^ Overcoming natural barriers for precise targeting of extrahepatic delivery remains a critical challenge for LNPs.^[^ ^158^, ^169^, ^170^ ^]^

## 5 Applications of Sonogenetics in Chronic Diseases

The advancement of mechanosensitive channels and gene delivery technologies has facilitated the emergence of sonogenetics, presenting new therapeutic possibilities for a range of diseases. Sonogenetics involves the targeted delivery of mechanosensitive protein genes to specific cells, followed by the activation of mechanosensitive channels using the mechanical properties of ultrasound. This activation induces changes in ion concentrations within and around the targeted cells in the ultrasound focal area, resulting in therapeutic outcomes.^[^ ^42^ ^]^ This innovative and non‐invasive approach to cell manipulation represents a significant advancement over sonogenetics.^[^ ^171^ ^]^ Since the concept of sonogenetics was proposed, researchers have continuously attempted to apply various mechanosensitive channels to mammalian cells to achieve clinical conversion. In the context of various diseases, chronic conditions such as epilepsy, Parkinson's disease, vision restoration, and cancer typically require long‐term and precise treatment strategies, and sonogenetics has demonstrated significant advantages in these areas (**Table** **2**).^[^ ^172^ ^]^

**Table 2:** The application of sonogenetics toolkit in treatment of chronic diseases.

Chronic diseases	Mechanosensitive channels	Gene delivery systems	Mechanism	Ref.
Parkinson's Disease	mPrestin (N7T, N308S)	AAV	Elevated expression of neurotrophin Amelioration of dopaminergic neuron degeneration	[4]
	MscL‐G22S	AAV	Increased effective dopamine release	[87]
Visual restoration	MscL‐G22S	AAV	Activate cortical neurons in the primary visual cortex	[16]
Neoplasm	MscL‐I92L	nanoliposomes	Massive influx of calcium ions induces tumor cell apoptosis	[86]

### 5.1 Treating Neurodegenerative Disorder Parkinson's Disease

Neuroregulation technology has been instrumental in researching brain function and brain diseases, including deep brain stimulation, transcranial magnetic stimulation, drug delivery system, photogenetics and so on.^[^ ^22^, ^173^, ^174^ ^]^ However, these technologies have many limitations such as low spatial resolution and poor penetration. Moreover, their invasive nature requires the surgical implantation of fibers or electrodes, which can result in potential bodily harm.^[^ ^42^ ^]^ In contrast, Sonogenetics has gained attention from researchers due to its superior penetrability, precise focusing capabilities, and non‐invasive characteristics. Various studies have progressed from the regulation of neural and muscle activity to the treatment of neurodegenerative diseases. For instance, Ibsen et al conducted a study where they knocked out TRP‐4 in C. elegans and observed a significant reduction in reversals in response to ultrasound stimulation compared to wild‐type C. elegans. This study is the first to demonstrate ectopic expression of TRP‐4 in model animals, making this article is also considered the origin of sonogenetics.^[^ ^7^ ^]^

In addition to validation in invertebrates, the use of sonogenetics in clinical applications is crucial for mammalian cells as well. Ye et al introduced MscL‐I92L into primary cultured rat hippocampal CA1/CA3 neurons and found that the transfected cells could generate action potentials upon 0.25MPa ultrasound stimulation. The successful and precise modulation of neuronal excitability in vitro has laid the foundation for the further development of sonogenetics.^[^ ^17^ ^]^ The following year, Huang et al transfected Venus mPrestin (N7T, N308S) into the VTA brain region deep in the mouse brain. Under 0.5 MHz FUS irradiation, the mice that received the transfer showed significant expression of c‐Fos, indicating that ultrasound stimulation induced neural activity in the transfected mice, which was attributed to Prestin. This study fully elucidates that Prestin transfected into neurons in vivo enables mammalian cells to perceive ultrasound stimulation, indicating the potential of ultrasound for treating deep brain tissue in mammals.^[^ ^103^ ^]^ Subsequently, Duque et al also discovered hsTRPA1, a mammalian protein that can be successfully expressed in various human cells and is highly sensitive to ultrasound stimulation.^[^ ^62^ ^]^

In addition, Zhu et al discovered that the mechanosensitive ion channel Piezo1 is functionally expressed in various brain regions. They conducted experiments on ultrasound‐induced neuronal activity in vivo using the Piezo1 knockout (P1KO) mouse model, and found that P1KO significantly reduced the neuronal calcium response, limb movement, and electromyographic amplitude induced by the right motor cortex. These findings suggest that sonogenetics could effectively regulate muscle movement, and the spatial distribution of Piezo1 in the brain is important for more precise regulation techniques.^[^ ^65^ ^]^

**Figure 10:**
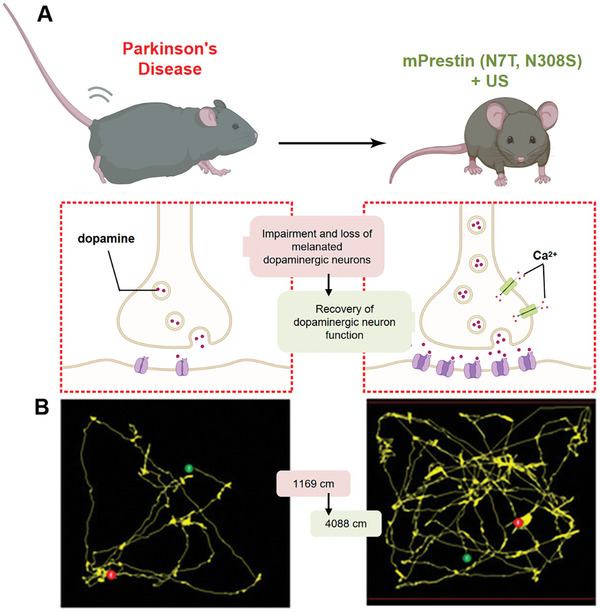
The improvement of motor function in Parkinson's disease mice by sonogenetics. A) Ultrasound stimulation increased effective dopamine release.^[^ ^87^ ^]^ B) Sonogenetics stimulation improved motor function recovery of PD mice.^[^ ^4^ ^]^ Reproduced with permission.^[^ ^4^ ^]^ Copyright 2021, American Chemical Society. Created with BioRender.com.

The above series of results indicate that sonogenetics has the ability to accurately activate specific neural circuits to elicit specific behavioral responses, which is consistent with the treatment of neurodegenerative diseases such as Parkinson's disease. In 2023, Qiu et al conducted a study to assess the efficacy of sonogenetics in activating specific neuronal activity and controlling behavior in free‐moving mice. They expressed MscL‐G22S in three groups of mice: in the right dorsal striatum (DMS), in the reward center region of the midbrain (VTA), and in the subthalamic nucleus (STN) neurons of PD mice. By using a wearable ultrasound transducer to deliver low‐intensity ultrasound stimulation, the researchers observed increased activity in DMS mice, synchronized release of dopamine in VTA mice in response to ultrasound stimulation, and relief in motor disorders in PD mice after MscL transfection. These findings suggest that sonogenetics can effectively modulate specific behaviors in freely moving mice by activating established neural circuits. Moreover, the study demonstrated that low‐frequency ultrasound could improve motor disorders in PD mice, hinting at the potential clinical applications of sonogenetics in treating neurodegenerative diseases (**Figure** **10**A).^[^ ^87^ ^]^ Fan et al reached a similar conclusion. They examined the expression of brain‐derived neurotrophic factor (BDNF) and nerve growth factor (NGF) 56 days after gene transfection, and western blot data suggested that repeated neuronal stimulation would upregulate BDNF and NGF expression and potentially activate BDNF/NGF‐facilitated synaptic plasticity. Further IHC staining of the dopaminergic neuron regions in PD mice revealed an improvement in dopaminergic neuron degeneration after repeated neuron stimulation. Finally, they used the beam‐walking and open field tests to examine the motor function of PD mice, and found that the receipt of Venus‐mPrestin (N7T, N308S) + US treatment could significantly improve the locomotor ability and the motor activity of PD mice. These findings suggest a promising therapeutic approach for PD patients in the future (Figure 10B).^[^ ^4^ ^]^

### 5.2 Restoring Vision

The tremendous success of sonogenetics in neural activity regulation has sparked interest in its potential applications for treating various diseases. However, restoring vision through ultrasound is not a straightforward task. Activating retinal ganglion cells with ultrasound stimulation requires high spatiotemporal resolution to effectively produce visual perception on the retina.^[^ ^175^ ^]^ The spatial precision of ultrasound stimulation directly impacts the quality of retinal imaging, while the temporal resolution of the stimulation determines the achievable visual frame rate.^[^ ^176^, ^177^ ^]^ This complexity makes it more challenging than typical ultrasound stimulation. Current research on neural regulation typically utilizes low‐frequency ultrasound to penetrate the skull and target deep neurons, resulting in limited spatial resolution (>3 mm) and delayed cell response to stimulation.^[^ ^178^ ^]^ This hinders the ability to target specific cells effectively. Visual restoration, a form of neural regulation, requires high temporal and spatial resolution, as well as acoustic intensity, to transmit intricate spatial patterns at a frequency of 13 Hz.^[^ ^179^ ^]^ Increasing ultrasound frequency to enhance spatial resolution is crucial, but studies have shown that excessively high frequencies, such as 30 MHz, can inhibit regulation.^[^ ^180^ ^]^ Moreover, elevating ultrasound intensity may lead to tissue damage from heat generation.^[^ ^181^ ^]^

**Figure 11:**
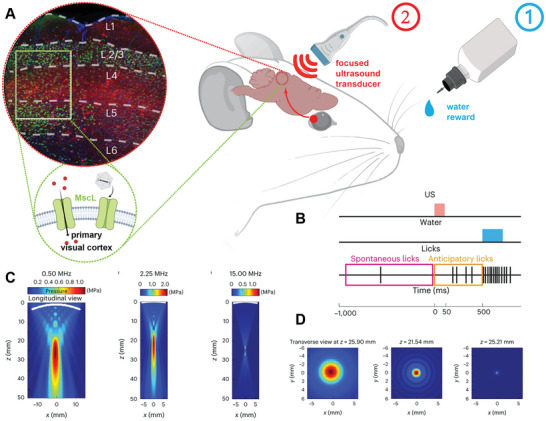
The role of sonogenetics in the primary visual cortex and activation of behavioral responses in mice following associative visual training. A,B) Behavioral response induced by the ultrasound stimulation in mice V1 cortex following visual training.^[^ ^16^ ^]^ C,D) Focal area for different ultrasound frequencies.^[^ ^16^ ^]^ Reproduced with permission.^[^ ^16^ ^]^ Copyright 2023, Springer Nature. Created with BioRender.com.

To date, several researches have explored ultrasound‐based activation strategies for visual restoration. For example, Jiang et al. designed a retinal piezoelectric array that can be stimulated by ultrasound, where each piezoelectric element can be individually activated.^[^ ^182^ ^]^ The study explored the effects of different ultrasound frequencies and intensities on the activation of retinal neurons and found that optimizing ultrasound frequency and intensity parameters can significantly improve the response rate of retinal neurons, ensuring effective optic nerve regulation. Additionally, Cadoni et al. highlighted how improvements in ultrasound equipment and gene delivery methods can achieve more precise and efficient retinal stimulation.^[^ ^16^ ^]^ They utilized multielectrode arrays to establish connections between neuronal cells and in vitro electronics, creating brain‐machine interfaces for detecting potential neuronal changes following ultrasound stimulation. G22S‐MscL gene was introduced into rat retinal ganglion cells in vitro and cortical neurons in the primary visual cortex in vivo using AAV. An ultrasound transducer was surgically attached to the outer surface of the dura mater via craniotomy, enabling consistent focal length ultrasound stimulation. The experimental results indicate that ganglion cells or visual cortex neurons respond to 10 ms of short duration or 10 Hz of fast frequency ultrasound stimulation. As the ultrasound frequency increases from 0.50 to 15.00 MHz, the depth at which the sound beam reaches its maximum intensity remains consistent with the focal length, while FWHM decreases to an initial 1/16 (Figure 11C,D). Meanwhile, the study observed a decrease in the range of cells activated by 15MHz ultrasound, but noted a significant increase in the density of activated cells. Using MEA, the researchers were able to clearly visualize the activated cells and determined that a transducer displacement of 0.4mm corresponded to a detected displacement of activated cells of 0.29 ± 0.09 mm. Anticipatory licking behavior was trained in water‐restricted mice through water supply after illumination (**Figure** **11**A,B). Results from ultrasound stimulation revealed that G22S‐MscL rats exhibited a higher number of anticipatory licks compared to NT rats following ultrasound stimulation. The above experimental results indicate that the sensitivity of retinal or cortical neurons to ultrasound increases after expressing G22S‐MscL. Under ultrasound activation at a frequency of 15 MHz, cells exhibited a millisecond latency and a spatial resolution of <400 µm, and generated behavioral motor responses similar to light perception through neuronal activation, which were not observed in the retina of blind mice or those using synaptic blockers.

### 5.3 Inhibiting Tumor Growth

Ultrasound regulation of mechanosensitive channel opening can cause changes in ion concentration inside and outside the cell membrane. This regulation not only impacts cell function but also has the potential to induce cell apoptosis, a key target in tumor treatment. Apoptosis plays a vital role in preventing tumor formation by eliminating abnormal cells. Tumor cells that resist apoptosis can develop resistance to therapy.^[^ ^183^ ^]^ As a result, targeting molecules involved in apoptosis has become a prominent area of research in tumor therapy. Various methods, such as chemical drugs and high temperatures, have been utilized to induce tumor cell apoptosis.^[^ ^184^, ^185^, ^186^, ^187^ ^]^ However, these methods face the problem of lacking precise regulation of tumor cell death, which can lead to non‐specific cell death and systemic side effects.^[^ ^188^ ^]^ For example, chemotherapy and radiotherapy, although effective in killing cancer cells, often lack precise targeting of tumor cells.^[^ ^189^ ^]^ This means that these treatments not only attack cancer cells but also damage surrounding healthy cells, leading to severe side effects such as nausea, fatigue, immune system suppression, and organ damage.^[^ ^190^ ^]^ Additionally, high‐temperature treatments, such as high‐intensity focused ultrasound, can also cause thermal damage to healthy tissues, further increasing the risks associated with the treatment process.^[^ ^191^ ^]^ Consequently, the effectiveness and broader application of these traditional methods are limited.

**Figure 12:**
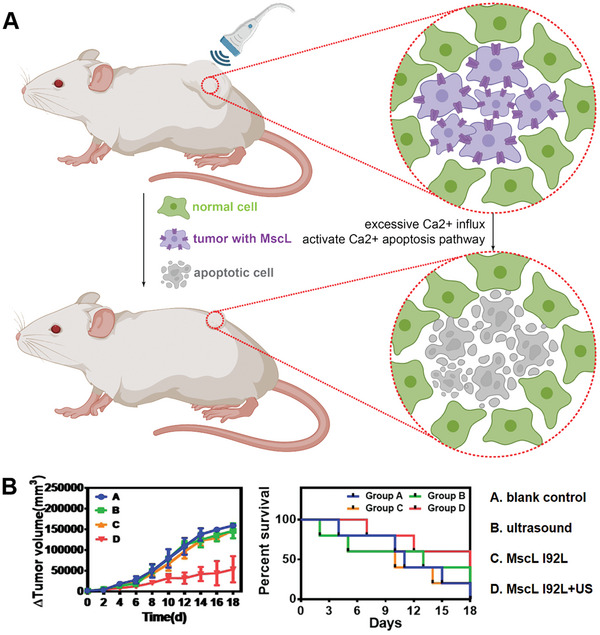
Schematic diagram of sonogenetics system for inhibiting tumor growth. A) Diagrammatic sketch of sonogenetics induced cell apoptosis. B) Function verification of the logic AND‐gated sonogenetics system in mice.^[^ ^86^ ^]^ Reproduced with permission.^[^ ^86^ ^]^ Copyright 2020, American Chemical Society. Created with BioRender.com.

In this context, sonogenetics, as an emerging therapeutic approach, holds the potential to reduce nonspecific cell death and systemic side effects. Recent years, in order to reduce tissue damage, Wang et al proposed a method in 2020 to accurately induce tumor cell apoptosis through sonogenetics.^[^ ^86^ ^]^ They developed a logic AND‐gated nanosystem that utilized cationic nanoliposomes to express the MscL‐I92L channel protein on tumor cell membranes, enhancing cell sensitivity to ultrasound stimulation. By keeping the MscL‐I92L protein channel open under continuous ultrasound stimulation, an excess of Ca^2+^ influx was achieved, leading to cell apoptosis (**Figure** **12**A). Targeted expression of channel proteins in tumor cells effectively minimized non‐specific triggering, thereby reducing side effects. The study found that the B16 cell line had the highest apoptosis rate (11.9% early apoptosis and 59.1% late apoptosis) among the tumor cell lines (HeLa, B16, and 4T1) after continuous 6MHz ultrasound irradiation for 30 min. These findings suggest that sonogenetics may hold promise as a novel approach for tumor treatment (Figure 12B).

However, despite the significant potential of sonogenetics in inhibiting tumors, further research is still needed to ensure its long‐term safety and effectiveness, which is also necessary for other chronic diseases.^[^ ^192^ ^]^ First, ultrasound waves of different frequencies, intensities, and durations are used in the process of sonogenetics, and their long‐term effects on different types of healthy tissues and tumor cells need to be clarified.^[^ ^193^ ^]^ Second, precise targeting of tumor cells by gene delivery systems is also crucial, as it can reduce non‐specific damage.^[^ ^194^ ^]^ Finally, the optimization of ultrasound equipment is also beneficial for precise control of the ultrasound action site.^[^ ^195^ ^]^

## 6 Conclusion

Ultrasound is a mechanical wave that can penetrate opaque materials and has mechanical effects.^[^ ^196^ ^]^ As the frequency of the ultrasound wave increases, the wavelength also becomes shorter, allowing for increased penetration but potentially sacrificing some spatiotemporal resolution.^[^ ^103^, ^197^ ^]^ Compared to other cell regulatory technologies, sonogenetics technology has good non‐invasive, convenient in vitro regulatory focusing, as well as spatiotemporal controllability and accuracy.^[^ ^35^, ^178^, ^198^, ^199^ ^]^ This has led to a growing interest in sonogenetics for therapeutic and interventional purposes. Key components for successful sonogenetics include mechanosensitive channels and gene delivery systems. **Table** **3** shows the developmental trend of sonogenetics research over time. Initial studies primarily concentrated on simple model organisms, utilizing relatively singular mechanosensitive channels and gene delivery systems. As research progressed, the focus gradually shifted to more complex mammalian cells and tissues, employing more diverse sonogenetics toolkit. Meanwhile, due to the varying responses of different mechanosensitive channels to various ultrasound frequencies, durations, and intensities, ultrasound parameters become more complex and precise to achieve specific regulation of various cellular activities. For example, the TRP‐4 channel is activated at a frequency of 2.25 MHz, while the MscL‐G22S channel is more sensitive to ultrasound stimulation and only requires a frequency of 0.5 MHz. The duration of ultrasound stimulation also affects channel activation; longer durations can more effectively activate the mPrestin channel, while shorter durations are suitable for the MscL‐G22S channel. We also observe that, although there is a variety of gene delivery systems available, many studies in the field of sonogenetics for chronic disease tend to favor AAV. This is likely due to virus based delivery vectors offering longer durations of gene expression and greater stability, which aligns with the long‐term, sustained gene expression required for chronic disease treatment. In contrast, while non‐virus based delivery vectors offer higher safety, the duration of gene expression is relatively shorter, with lower expression efficiency and stability. As a result, lipid‐based systems are more suitable for short‐term gene regulation and often require regular, repeated treatments to maintain therapeutic efficacy. These findings enable further researches to optimize ultrasound parameters based on specific channel types and application requirements, thereby maximizing mechanical effects and achieving the most effective biological functions.

**Table 3:** Evolution and progress of sonogenetics.

Study	Mechanosensitive channel	Gene delivery system	Target species	Ultrasound parameters			Regulation
				Frequency [MHz]	Duration [ms]	Intensity [MPa]	
Ibsen et al. (2015)	TRP‐4	/	*C. elegans*	2.25	10	0 – 0.9	behavior of *C. elegans*
Ye et al. (2018)	MscL I92L	lentivirus	rat hippocampal CA1/CA3 neurons	1 – 5	50	0.12 – 0.45	neuronal activities
Huang et al. (2019)	mPrestin (N7T, N308S)	AAV	HEK293T cells	0.08 – 3.5	3000	0.5	calcium influx
Qiu et al. (2020)	MscL‐G22S	AAV	HEK293T cells; mouse embryonic cortices	0.5	300	0.05 – 0.5	calcium influx neuronal activities
Wang et al. (2020)	MscL I92L	nanoliposomes	tumor cell lines	2 – 12	5 – 30 min	0 – 5	tumor apoptosis
Wu et al. (2020)	mPrestin (N7T, N308S)	nanoliposomes	SH‐SY5Y cells	0.5	3000; 1 min	0.1 – 0.5	calcium influx electrophysiology
Fan et al. (2021)	mPrestin (N7T, N308S)	AAV	SH‐SY5Y cells; PD mice dopaminergic neurons	0.5	3000	0.5	membrane potential; functional behavioral recovery in PD mice
Liao et al. (2021)	Piezo1	plasmid	HEK293T‐P1KO cells	shear stress in target area: 18 – 74 dyne/cm^2^			highest calcium influx
Sorum et al. (2021)	TRAAK K+	plasmid	xenopus laevis oocytes	5	10	/	channel activation
Duque et al. (2022)	191 candidates; hsTRPA1	AAV	HEK293T cells; mouse primary embryonic neuron	range; 7	1 – 100	1 – 2.5	calcium influx; electrophysiology; neuronal activities
Vasan et al. (2022)	hsTRPA1	AAV	HEK293T cells	1 – 10	100	0.2 – 0.6	neuronal activities
Zhou et al. (2022)	MEC‐4 and MEC‐6	/	*C. elegans*	27.4	6.4	3	behavior of *C. elegans*
Cadoni et al. (2023)	MscL‐G22S	AAV	rat retinal ganglion cells; cortical neurons of the primary visual cortex	0.5, 2.25, 15	10 – 200	0.2 – 1.4	visual restoration
Xu et al. (2023)	TRPV1	AAV	neurons of the M2 cortex	1.5	15 000	0, 0.7, 1.1	neuronal activities behavior of mice
Zhu et al. (2023)	Piezo1	AAV	neurons in different brain regions	0.5	50, 250, 500	0 – 0.8	neuronal activities limb movement
Xian et al. (2023)	MscL‐G22S	AAV	neurons in the dorsal striatum	0.5, 0.9	300	0.05 – 0.7	neuronal activities locomotion of mice

In specific applications, ultrasound concentrates its maximum sound intensity near the focal point, triggering the opening of mechanical sensitive channels on target cells through gene delivery vectors in the focal area. This process leads to ion exchange inside and outside the cell, initiating intracellular signaling pathways that can modulate cell activities such as proliferation, differentiation, and apoptosis.^[^ ^12^, ^18^, ^197^ ^]^ As a consequence, it has the ability to play a significant role in neural regulation, visual recovery, tumor suppression, and other aspects. The unique advantages of sonogenetics have attracted the attention of researchers, but there are still many urgent problems that need to be solved in this technology. First, the mechanism by which sonogenetics produces biological effects is not yet clear. Proposed mechanisms include heating, cavitation, separating the leaves of life members, or affecting the activity of mechanosensitive ion channels.^[^ ^37^, ^198^, ^200^, ^201^, ^202^ ^]^ Currently, ultrasound is widely recognized as a mechanical wave that acts on mechanosensitive channels, leading to biological effects.^[^ ^18^ ^]^ Thermal and cavitation effects are also involved in ultrasound regulation.^[^ ^200^, ^203^, ^204^ ^]^ Enhancing the accuracy of spatiotemporal resolution in ultrasound regulation remains a challenge, with ongoing efforts in the literature to address this issue while prioritizing safety.^[^ ^12^ ^]^ Sonogenetics involves delivering genes of mechanosensitive proteins to targeted cells in the human body. Current gene delivery systems rely on virus delivery, but their immunogenicity raises safety concerns.^[^ ^205^ ^]^ Screening for mechanically sensitive channels early on is crucial, as some ion channels respond to ultrasound in nematodes or primary cells but not in mammals.^[^ ^7^, ^62^ ^]^ While research focuses on activating target cells, there is limited investigation into proteins that inhibit target cells. Addressing these issues is essential for precise regulation of target cells.^[^ ^6^, ^8^, ^64^ ^]^ Early ultrasound limitations included limited application in higher organisms due to the use of microbubbles.^[^ ^7^, ^206^, ^207^ ^]^ Mutants of mechanically sensitive channels have increased sensitivity to mechanical stimulation.^[^ ^17^, ^64^, ^208^ ^]^ Stable ultrasound stimulation using wearable devices is key for clinical applications, but further research is needed in this area. Lastly, long‐term safety and effectiveness require the accumulation of large‐scale preclinical studies and clinical trials, which require precise coordination from various fields such as medicine, engineering, basic medicine, and relevant institutions to jointly address major issues in human health.

Despite ongoing challenges, sonogenetics technology has advanced significantly due to increased research efforts. Researches in sonogenetics have gradually transitioned from in vitro experiments to in vivo experiments, successfully extending from invertebrates to mammals. This demonstrates the potential of sonogenetics in complex organisms and lays the groundwork for further clinical applications. Meanwhile, sonogenetics, as a non‐invasive technology, provides patients with a gentler and more effective treatment option.^[^ ^19^ ^]^ Therefore, it mainly offers a new avenue for treating chronic diseases such as Parkinson's disease, vision restoration, and cancer.^[^ ^4^ ^]^ At the same time, the successful activation of brain cells and heart cells expressing mechanical sensitive channel proteins has also given researchers hope for the clinical transformation of sonogenetics.^[^ ^18^ ^]^ Similar to deep brain stimulation, technologies such as cardiac pacemakers and insulin pumps that require the activation of specific cells will also usher in minimally invasive or non‐invasive activation through sonogenetics, which will be a revolutionary change.^[^ ^209^ ^]^ Future developments will involve more in‐depth large‐scale experiments in primates to comprehensively understand the effects of sonogenetics within living organisms.

**Figure 13:**
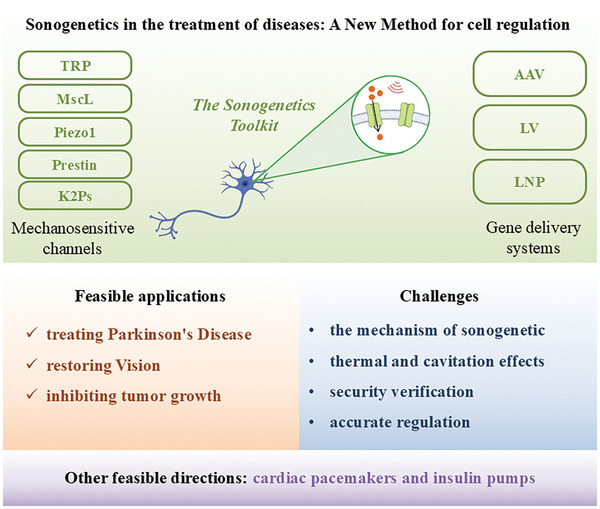
A summary of the sonogenetics toolkit, its applications in chronic diseases such as Parkinson's disease, vision impairment, and cancer, and the challenges in optimizing gene delivery systems and mechanosensitive channels.

The novelty of this review lies in its detailed exploration of the specific applications of sonogenetics in the treatment of chronic diseases, addressing gaps in the existing literature. It enables readers to better understand the tremendous potential of sonogenetics in addressing chronic conditions such as Parkinson's disease, vision restoration, and cancer, as well as the current technical challenges and promising prospects in this field (**Figure** **13**).

## Conflict of Interest

The authors declare no conflict of interest.
